# A novel human *ex vivo *model for the analysis of molecular events during lung cancer chemotherapy

**DOI:** 10.1186/1465-9921-8-43

**Published:** 2007-06-14

**Authors:** Dagmar S Lang, Daniel Droemann, Holger Schultz, Detlev Branscheid, Christian Martin, Anne R Ressmeyer, Peter Zabel, Ekkehard Vollmer, Torsten Goldmann

**Affiliations:** 1 Clinical and Experimental Pathology, Research Center Borstel, D-23845 Borstel, Germany; 2 Medical Clinic, Research Center Borstel, D-23845 Borstel, Germany; 3 Department of Thoracic Surgery, Hospital Großhansdorf, D-22927 Großhansdorf, Germany; 4 Division of Pulmonary Pharmacology, Research Center Borstel, D-23845 Borstel, Germany; 5 Medical Clinic III, University of Schleswig-Holstein, Campus Lübeck, D-23538 Lübeck, Germany

## Abstract

**Background:**

Non-small cell lung cancer (NSCLC) causes most of cancer related deaths in humans and is characterized by poor prognosis regarding efficiency of chemotherapeutical treatment and long-term survival of the patients. The purpose of the present study was the development of a human *ex vivo *tissue culture model and the analysis of the effects of conventional chemotherapy, which then can serve as a tool to test new chemotherapeutical regimens in NSCLC.

**Methods:**

In a short-term tissue culture model designated STST (Short-Term Stimulation of Tissues) in combination with the novel *HOPE-fixation and paraffin embedding method we examined the responsiveness of 41 human NSCLC tissue specimens to the individual cytotoxic drugs carboplatin, vinorelbine or gemcitabine. Viability was analyzed by LIFE/DEAD assay, TUNEL-staining and colorimetric MTT assay. Expression of Ki-67 protein and of BrdU (bromodeoxyuridine) uptake as markers for proliferation and of cleaved (activated) effector caspase-3 as indicator of late phase apoptosis were assessed by immunohistochemistry. Transcription of caspase-3 was analyzed by RT-PCR. Flow cytometry was utilized to determine caspase-3 in human cancer cell lines.

**Results:**

Viability, proliferation and apoptosis of the tissues were moderately affected by cultivation. In human breast cancer, small-cell lung cancer (SCLC) and human cell lines (CPC-N, HEK) proliferative capacity was clearly reduced by all 3 chemotherapeutic agents in a very similar manner. Cleavage of caspase-3 was induced in the chemo-sensitive types of cancer (breast cancer, SCLC). Drug-induced effects in human NSCLC tissues were less evident than in the chemo-sensitive tumors with more pronounced effects in adenocarcinomas as compared to squamous cell carcinomas.

**Conclusion:**

Although there was high heterogeneity among the individual tumor tissue responses as expected, we clearly demonstrate specific multiple drug-induced effects simultaneously. Thus, STST provides a useful human model to study numerous aspects of mechanisms underlying tumor responsiveness towards improved anticancer treatment. The results presented here shall serve as a base for multiple functional tests of novel chemotherapeutic approaches to NSCLC in the future.

*Hepes – Glutamic acid buffer mediated *O*rganic solvent *P*rotection *E*ffect

## Background

To date, no effective chemotherapeutic treatment for non-small cell lung cancer (NSCLC) exists [1,2]. Therefore, this type of tumor is characterized by a poor prognosis with regard to clinically successful chemotherapy and long-term survival of the patients [3]. Little is known about the complex interactions taking place within the human lung upon chemotherapy. One reason for this might be implied by the common models used for analyzing such interactions like cell cultures or animal models, since such experimental data can be transferred only to a limited extent. As part of a large scaled investigation aimed at improving the facilities available today for the treatment of NSCLC, we report the use of an *ex vivo *tissue culture model (STST: Short-Term Stimulation of Tissues) [4,5] in combination with the novel HOPE-technique (*H*epes – Glutamic acid buffer mediated *O*rganic solvent *P*rotection *E*ffect) [6] to gain insight into the cellular events taking place upon conventional chemotherapy.

Such *ex vivo *models are long known [7], however; this technique up to date has failed to become widely used in clinical sciences. The major reason for this is due to the fixation of tissues with formalin; although morphology is well maintained, the application of molecular techniques is largely restricted due to degradation of nucleic acids and protein cross-linking. Since drug-induced effects or immunological reactions within the tissue are hardly correlated with morphological but with molecular changes, the application of a better suited fixation technique allowing for molecular read outs would be a step ahead. With the development of the novel HOPE-technique, immunohistochemical detection [8] has been considerably improved and together with excellent preservation of nucleic acids, molecular analyses can be comprehensively applied [5,9-12]. The combination of short-term cultivation using vital tissues and HOPE-fixation (STST) has already been described for other functional studies in the human system [4,5,13,14]. To date, there is only one description on the behavior of NSCLC in organ culture, which was based on formalin-fixed, paraffin-embedded specimens and included only a limited number of tissues with no comprehensive molecular read out [15].

With regard to the high cellular heterogeneity of NSCLC [16], experimental data are necessary to elucidate the molecular mechanisms underlying tumor behaviour in detail, thus providing the base for the development of individual and more efficient anticancer treatment regimens. However, most experimental data are based on either animal models, the extrapolation of which to humans is limited or on cell lines that cannot mimic both the complexity and heterogeneity of tumor tissues. As a consequence, we hypothesize that the use of vital human lung tumor tissue specimens would provide a promising novel *ex vivo *model to elucidate the molecular mechanisms underlying tumor behavior in detail, thus providing the base for the development of individual and more efficient anticancer treatment regimens. Furthermore, such a model, in contrast to cell culture, enables to study the influence of inflammatory cells, which can make up a substantial part of the tumor scenario.

In order to evaluate the suitability of this novel short-term *ex vivo *model (STST) using human NSCLC specimens, we studied possible drug-induced alterations of multiple known relevant biomarkers for human NSCLC [6]. First, the effects of the chosen culture conditions on the viability of tumor tissues were assessed by LIVE/DEAD viability/cytotoxicity assay using 2-photon microscopy in two separate experiments. To analyze the effects of conventional chemotherapy in STST, each of the chemotherapeutic agents including carboplatin, vinorelbine and gemcitabine, was directly applied *ex vivo *to 41 different human lung tumor specimens of both squamous cell – and adenocarcinoma type for a 16 h culture period. These anticancer drugs are widely used for treatment of NSCLC patients. They prevent cell proliferation by DNA damage or by tubulin disintegration and also inhibit cellular repair mechanisms. Vinorelbine is also known to inactivate bcl-2 by phosphorylation, thus initiating apoptosis [17].

A series of cell culture experiments using A549 (NSCLC, adenocarcinoma type), CPC-N (SCLC, small-cell lung carcinoma), HeLa, and HEK cell lines was performed under identical chemotherapeutical culture conditions to compare our results with those obtained by an established experimental model regarding both viability and functionality of the cells. Furthermore, specimens of breast cancer were also treated like the lung tumor samples to verify the efficiency of the used drug concentrations on the chosen biomarkers in a chemo-susceptible type of cancer.

After cultivation, tissue samples were fixed using the novel HOPE technique and paraffinized as described elsewhere [5]. After deparaffinization, protein expression of Ki-67 as indication for the proliferative fraction of the tumour cells was assessed by immunohistochemistry. BrdU uptake as a marker for DNA synthesis in activated cells of S phase was also included as test of more functional relevance. An important key regulator of the apoptotic pathway such as caspase-3 was also evaluated immunohistologically to study the induction of apoptosis. For the cell lines, drug-induced expression of caspase-3 was analyzed by flow cytometry. In a limited number of experiments, analyses of specific mRNA of caspase-3 were also performed by reverse transcriptase – polymerase chain reaction (RT-PCR) in order to verify the results obtained on the protein level. In addition, we exemplarily assessed drug-induced DNA strand breaks in apoptotic cells by the TUNEL labelling assay, to further validate the importance of cleaved caspase-3 as a relevant biomarker for apoptosis.

In an ideal setup these data would have been correlated to actual patient responses to treatment; however, not all of the patients subjected to lung surgery will receive chemotherapy. Moreover, these chemotherapeutic interventions usually do not take place in the moment directly after surgery. Nevertheless, such data – if available – will be collected on the long-run.

## Methods

### Lung cancer tissues

Tumor samples were obtained from 42 patients (31 males, 11 females) undergoing lobe- or pneumectomy because of lung cancer, their age ranging between 43 and 78 years. A total of 41 vital non-small cell lung cancer (NSCLC) specimens were tested including 21 adenocarcinomas (AC), 20 squamous cell carcinomas (SCC), all of them with differentiation grades 2 or 3, except 2 squamous cell carcinomas and 1 adenocarcinoma with grade 1. One sample of small-cell lung carcinoma (SCLC) was included as chemo-sensitive type of lung cancer. 

Four different biopsy samples of breast cancer (classifications: T2, G2-3; oestrogen receptor IRS: 0,2,12,12; progesterone receptor IRS: 0,2,4,6; Hercept Score: 0,0,0,3+) from female patients were also cultivated and treated identically to the lung cancer tissue specimens to compare drug-induced effects in a different, more chemo-susceptible type of cancer with those in NSCLC. 

### Cultivation and chemotherapeutical treatment of human cancer tissues

Four different biopsy samples of breast cancer, 1 sample of human SCLC and 41 specimens of human NSCLC were cultivated as previously reported [4]. Shortly, vital specimens were cultured in 2 ml RPMI 1640 at 37°C and 5% CO_2 _for 16 h in the presence or absence of the individual chemotherapeutic agents including carboplatin, vinorelbine or gemcitabine. These cytotoxic drugs are widely used for treatment of human NCSCL carcinomas. Their final concentrations of 8.25 μg/ml, 0.76 μg/ml and 0.31 μg/ml, respectively, were calculated based on common human dose regimens.

### Viability

To visualize cell viability in slices of exemplary NSCLC tissue specimens, 2-photon microscopy was used in combination with the LIVE/DEAD^® ^viability/cytotoxicity assay kit (Molecular Probes, Eugene, Oregon, USA). The fluorescent dyes were excited at 800 nm with a Ti:Sa femtosecond laser (Coherent, Dieburg, Germany). Slices were analyzed directly after preparation and 16 h later in the presence and absence of the different cytotoxic drugs. To visualize the total amount of dead cells, the slices were treated with 1% Triton X-100 for 20 min prior to incubation with dyes. Six different areas in each tissue sample were evaluated and the results are expressed as percentage of dead cells with Triton X-100 values as 100%.

### BrdU uptake

Twenty eight tissue samples (19 AC, 9 SCC) were treated simultaneously with 5 μM BrdU (5-bromo-2'-deoxyuridine, Sigma-Aldrich, Steinheim, Germany) for 16 h to analyze the ongoing capacity of novel DNA-synthesis within tumor specimens under the different treatment conditions.

### Immunohistochemistry (IHC)

Tissue samples were fixed by the HOPE-technique and embedded in paraffin as described elsewhere [6]. Biomarkers (Ki-67, BrdU, cleaved caspase-3) were studied by IHC as published earlier [8,18].

Primary antibodies MIB-1 (Dako, Glostrup, Denmark [19], 333 ng/ml), monoclonal mouse-anti BrdU antibody (Dako, Glostrup, Denmark) and polyclonal rabbit antibody against human caspase-3 (cleaved) (DCS, Hamburg, Germany) were used in final dilutions of 1:100, 1:30 and 1:200, respectively. After 30 min (MIB-1), 45 min (caspase-3, cleaved) or 1 h (BrdU) at room temperature, visualization was performed by horseradish-peroxidase labeled streptavidin-biotin technique (LSAB2™, Dako, Denmark) diluted 1:3 for all antibodies and using 3-Amino9-Ethylcarbazole/H_2_O_2 _as chromogen. Slides were counterstained with Mayer's hemalum and mounted with Kayser's glycerinegelatine. Negative controls were included omitting the respective primary antibodies.

### Reverse transcriptase – polymerase chain reaction (RT-PCR)

In 5 different tissue culture experiments, RT-PCR was additionally performed as described elsewhere [5] using caspase-3 specific primers (forward 5'TGTTCTAAA-GGTGGTGAGGC; reverse 5'GTCTAGAGTCCTATGTGCTC) spanning an amplicon of 192 bp. Specific primers targeting the mRNA of the housekeeping gene glyceraldehyde – 3 phosphate dehy-drogenase (GAPDH) (forward 5'AGAACGGGAAGCTTGTCATC; reverse 5'TGC-TGATGATCTTGAGGCTG) spanning an amplicon of 247 bp were always run in parallel for reasons of control. RT-PCR products of caspase-3 were normalized to those of GAPDH for direct comparisons between the different treatment conditions.

### *In situ *cell death detection (TUNEL labeling assay)

For the detection and quantification of DNA strand breaks in apoptotic cells in exemplary NSCLC tissue samples in response to the different cytotoxic drugs, an *in situ *cell death detection kit AP (Roche Applied Science, Germany) was used. Sections of deparaffinized specimens were permeabilized for 5 min at RT. The addition of TUNEL-reaction mixture and subsequent visualization was performed according to the manufacturer's instructions using a reaction time of 1–2 min at RT for the chromogen (new-fuchsin).

### Cell culture and chemotherapeutical treatment *in vitro*

For cell culture, 0.5 × 10^6 ^A549 (NSCLC, AC) or HeLa (cervix carcinoma) cells and 1.0 × 10^6 ^HEK (kidney carcinoma) cells were transferred in 6 well plates. After a 24-hour plating period identical drug concentrations as described for the NSCLC samples were applied for 16 h at 35°C and 5% CO_2_or 6.3% CO_2_, respectively. CPC-N cells (SCLC) were split in half and cultivated in small culture flasks for 2 days before addition of the cytotoxic drugs. After termination of the culture, cells were centrifuged and fixed with HOPE reagent [12]. Caspase-3 expression was analyzed by IHC and by PE-conjugated active caspase-3 apoptosis kit I (BD PharMingen), except for CPC-N cells, in combination with fluorescence-activated cell sorter (FACS) analysis as described elsewhere [5].

### Cytotoxicity

Cytotxicity of the individual chemotherapeutic agents carboplatin, vinorelbine and gemcitabine was measured for each cell line after 16 of cultivation by the MTT (3-(4,5-dimethyldiazol-2-yl)-2,5-diphenyltetrazolium-bromid) colorimetric assay. The test is based on the ability of mitochondrial dehydrogenase in viable cells to convert MTT reagent (Sigma, Taufkirchen, Germany) into a soluble blue formazan dye. Briefly, The different cell lines were seeded into 96-well plates at a concentration of 0.125 × 10^6 ^cells/100 μl/well. After 24 h of plating period, the individual cytotoxic drugs were added at the same concentrations as indicated for the tumor specimen. At the end of the cultivation period, 10 μl of MTT reagent (5 mg/ml) were added and cell cultures were incubated for 4 h at 37°C. After removal of the culture medium, cells were lysed (isopropanol 0.04N HCL) to determine the amount of formazan product. Absorption was measured by a microplate reader (μ-quant, Bio-Tech Instruments) at 550 nm and the results were expressed as percent decrease of cell viability as compared to untreated controls.

### Statistical analysis

Results of viability testing and RT-PCR analyses as well as the data for human cell lines are shown as mean values ± SEM (mean of standard error). Median values ± SEM were used for breast cancer samples due to the high individual heterogeneity despite the low number of specimens. Statistical comparisons between treated NSCLC tissues and the respective untreated control tissue samples following short-term cultivation were performed for each cytotoxic drug separately, using nonparametric Mann-Whitney-test for unpaired samples (INSTAT, GraphPaD Software UNC, Chapel Hill, USA). Untreated cultured NSCLC tissue samples (medium controls) were also compared with fresh (native) tissue specimens to compare for cultivation effects upon the different biomarkers. A two-sided value of P ≤ 0.05 was considered significant.

## Results

### Effects of cultivation

#### Viability

**Figure 1:**
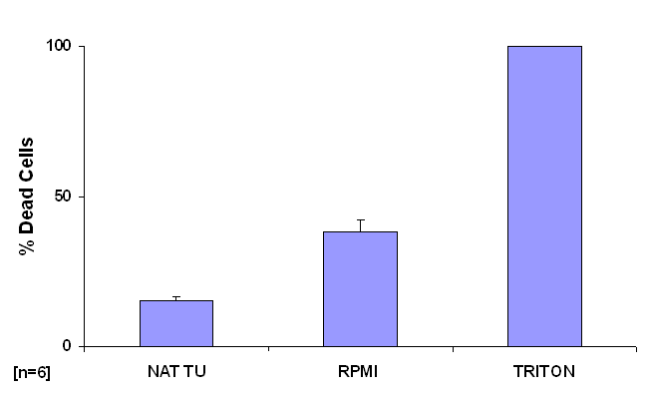
Percentages of dead cells in human NSCLC tissue specimens following 16 h culture period *ex vivo*. Viability was also assessed without cultivation (native tumor; NAT TU) to compare for culture effects. Data are expressed as mean percentage of dead cells + STD (standard deviation) (n = 6) with Triton X values as 100%.

The results are displayed in Fig. 1. Treatment of tissues with Triton X-100 resulted in elevated cell death (compared to native tissues) the amount of which was set at 100%. The fraction of dead cells was 15% in native tissues and increased to 38% at the end of cultivation.

#### *De novo *DNA synthesis

After termination of the 16 h culture period, 57% (16/28) of all tested NSCLC tumor specimens exhibited *de novo *DNA synthesis rates (measured by BrdU-incorporation) between ≥5% and 10% (3 specimens with 15–25%) of positive nuclear staining, confirming that tumor cells within the tissue remained vital and proliferative activity continued under the chosen culture conditions. Although not all of the NSCLC specimens were tested for this marker, untreated AC exhibited lower proliferation rates than SCC (AC n = 19, SCC n = 9). This becomes evident by the respective median values of 3.0% (AC) versus 5.0% (SCC).

#### Growth fraction

Without cultivation expression of Ki-67 was considerably lower in AC compared to SCC, as demonstrated by the mean values of positive nuclear staining in native tumors of 29.2% ± 3.3% (AC) versus 41.7% ± 4.0% (SCC), respectively (Table 1). Short-term cultivation of the NSCLC specimens (without chemotherapy) resulted in statistically significant reductions of Ki-67 protein with mean values of 18.7% ± 2.5% (AC) and 27.4% ± 2.5% (SCC).

**Table 1:** Effects of short-term cultivation upon expression of Ki-67 antigen and of cleaved caspase-3 expression in human NSCLC specimens of both adenocarcinoma and squamous cell carcinoma type. Data are expressed as mean values of percentages ± SEM.

**Ki-67**	**Tissue Type**		**16 h Culture**	**P-Value**	**Number of Tested Samples**
		**NAT TU**	**RPMI**		

	**AC**	29.2 ± 3.3	18.7 ± 2.5	**0.035**	N = 20
	**SCC**	41.7 ± 4.1	27.4 ± 2.5	**0.009**	N = 19
**Caspase-3**					
	**AC**	12.0 ± 2.9	11.1 ± 2.7	0.87	N = 18
	**SCC**	9.2 ± 4.3 (**median = 2.5)**	8.6 ± 1.3 (**median = 7.5**)	**0.018**	N = 17

AC : adenocarcinomas ; SCC : squamous cell carcinomas

NAT TU: native tumour (without cultivation); RPMI: cultivated tissues in medium

#### Apoptosis (cleaved caspase-3)

After cultivation AC exhibited protein levels of activated (cleaved) caspase-3 that were not significantly different from the respective native specimens (Table 1). SCC showed a statistically significant enhancement of expression of caspase-3 after cultivation that was evident in 73% (11/15) of those tissue samples with low levels of this protein. Two samples of the tested 17 SCC specimens, however, exhibited exceptionally high levels of cleaved caspase-3, ranging between 50% and 60% in the native tumor tissues, shifting the mean value above the corresponding mean value of the untreated cultured tumor samples. Therefore, the median values are also given in Table 1. The general expression levels of caspase-3 with or without cultivation were comparably low.

### Effects of chemotherapeutical treatment in chemo-sensitive cancer

#### Human breast cancer tissues

The expression of both Ki-67 antigen and cleaved caspase-3 was consistently altered in the presence of the individual chemotherapeutic agents when compared to RPMI-controls: Proliferation rates were decreased demonstrating median values of 15% ± 4.3% and 15% ± 5.5% of positive nuclear staining in the presence of vinorelbine and gemcitabine, respectively, as compared to 27.5% ± 7.8% in the medium control tissues (Table 2). Simultaneously, cleaved caspase-3 was increased about 2fold with median values of 25.0% ± 5.4% and 20.0% ± 6.6%, respectively, compared to 12.5% ± 3.1% in the untreated control tissues.

**Table 2:** Effects of chemotherapeutical treatment upon expression of Ki-67 and of Caspase-3 in four human breast cancer tissue specimens. Data are expressed as median values of percentages ± SEM of positively stained tumour cells.

**Ki-67**				**Two-tailed P-Value (n = 4)**
	**Treatment**		**RPMI**	

	**VIN**	15.0 ± 4.3	27.5 ± 7.8	0.770
	**GEM**	15.0 ± 5.5	27.5 ± 7.8	0.663
**Caspase-3**				
			**RPMI**	
	**VIN**	25.0 ± 5.5	12.5 ± 3.1	0.772
	**GEM**	20.0 ± 6.6	12.5 ± 3.1	0.559

RPMI: medium control tissues; VIN: vinorelbine; GEM: gemcitabine

Caspase-3 activation in human breast cancer is exemplarily displayed in the absence (**A**) or presence (**B**) of gemcitabine [see Additional files 1 and 6].

#### Human cell lines HeLa, HEK, chemo-responsive CPC-N and A549

Treatment of four established human tumor cell lines with chemotherapeutic drugs was performed for means of comparison to STST. Cultivation of these cell lines without chemotherapeutic agents resulted in a viability of all cell lines mostly above 90%, as determined by colorimetric MTT assay.

Upon treatment, vitality of HeLa and of A549 cells was reduced by 30% in response to vinorelbine (MTT assay); viability of the others remained nearly unaltered.

In CPC-N cells, which are derived from the chemo-sensible SCLC type of lung cancer, Ki-67 expression was consistently reduced by more than 50% in response to all three drugs; the same holds true for HEK cells (about 90% positively stained tumor cells in medium controls vs. 30 – 40% in the presence of the cytotoxic drugs). In contrast, the Ki-67-index of 90% in the chemo-resistant A549 cell line, derived from AC, remained nearly unaffected by the different chemotherapeutic agents; the same was observed in HeLa cells.

**Figure 2:**
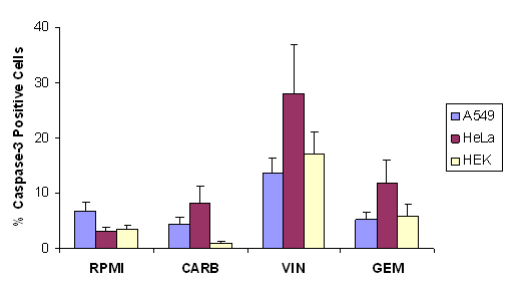
Caspase-3 induction determined by flow cytometry in human cancer cell lines in the absence (RPMI) or presence of carboplatin (CARB), vinorelbine (VIN) and gemcitabine (GEM), respectively. The cell lines were plated 24 h before addition of the single drug. Percentage of caspase-3 positive cells are shown as mean values + SEM (n = 4–5).

**Figure 3:**
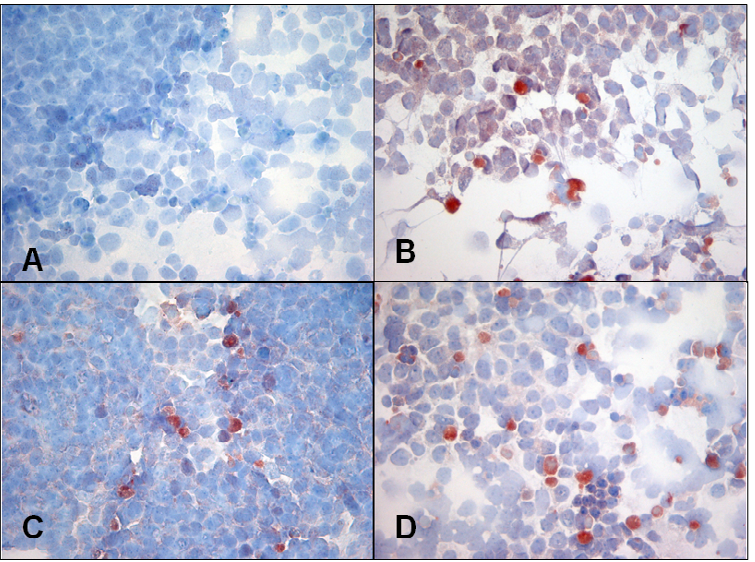
**A-D**: Induction of cleaved caspase-3 as determined by IHC in CPC-N cells (small-cell lung cancer) in the absence (**A**) or presence of carboplatin (**B**), vinorelbine (**C**) and gemcitabine (**D**), respectively (all 400×).

Caspase-3 induction, as determined by flow cytometry, was highly variable in response to carboplatin and gemcitabine (Fig. 2). Vinorelbine appeared to enhance caspase-3 expression more effectively than the two other drugs. Unfortunately, CPC-N cells showed drastically reduced viability to less than 20–30% upon resuspension, which is why data regarding caspase-3 expression had to be acquired by IHC. Here, CPC-N cells demonstrated negligible caspase-3 expression without treatment, but revealed consistent induction up to 30% in response to each cytotoxic drug (Figs. 3A–D).

In marked contrast, corresponding A549 cell preparations did not show effective caspase-3 protein expression following chemotherapeutical treatment by IHC. Accordingly, additional analyses at the mRNA level revealed that gene expression of caspase-3 in unresponsive A549 cells was not altered in the presence of cytotoxic drugs (not shown).

#### SCLC

**Figure 4:**
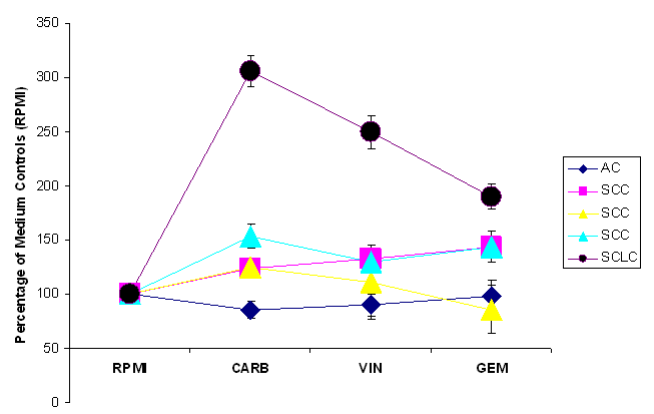
Transcription of caspase-3 in four different human NSCLC tissue specimens (n = 3 SCC; n = 1 AC) and one human SCLC (small-cell lung cancer) tissue sample in the absence (RPMI) or presence of carboplatin (CARB), vinorelbine (VIN) and gemcitabine (GEM), respectively. Signals of caspase-3 were normalized to the respective GAPDH signals and the resulting values of the untreated medium control tissues (RPMI) were set 100%. The data are shown for each individual specimen separately and are expressed as mean values ± SEM (n = 2). Lines were drawn through all data points derived from a single specimen to better reveal the individual trend of response.

One vital specimen of a patient with (chemo-sensitive) SCLC, which remarkably had been subjected to surgery, could also be analyzed by RT-PCR and IHC. In RT-PCR SCLC revealed consistently upregulated specific gene expression that was 3fold, 2.5fold and 2fold increased above control values in response to carboplatin, vinorelbine or gemcitabine, respectively (see Fig. 4). Accordingly, protein levels of cleaved caspase-3 were elevated by 25% in response to carboplatin and vinorelbine and to 15% in response to gemcitabine as compared to 3% within untreated control tissues (data not shown).

### Effects of chemotherapeutical treatment in non-responsive human NSCLC specimens

#### *De novo *DNA synthesis

BrdU staining patterns were heterogeneous among human NSCLC tissues (Table 3). In response to the individual anticancer agents, proliferative activity was always effectively abrogated in all NSCLC specimens mostly to less than 1% positively labeled cells within the tissues.

**Table 3:** Effects of chemotherapeutical treatment on expression of proliferation (Ki-67, BrdU) and of apoptosis (cleaved caspase-3) in human NSCLC tissue specimens. Data are expressed as mean values of percentages ± SEM of positively stained tumour cells.

**Ki-67**	**Treatment**			**Two-tailed P-Value**	**Number of Tested Samples**
**AC**			**RPMI**		

	**CARB**	9.8 ± 2.2	18.0 ± 2.6	**0.032**	N = 19
	**VIN**	10.9 ± 2.4	18.2 ± 2.4	**0.047**	N = 21
	**GEM**	11.4 ± 2.1	21.2 ± 2.1	**0.0049**	N = 17
**SCC**					
	**CARB**	19.1 ± 2.4	26.6 ± 2.4	0.065	N = 19
	**VIN**	18.1 ± 2.6	27.3 ± 2.4	**0.0026**	N = 20
	**GEM**	21.2 ± 2.9	28.8 ± 2.5	0.07	N = 17
**BrdU**					
**AC**			**RPMI**		
	**CARB**	0.9 ± 0.5	5.5 ± 1.7	**0.006**	N = 19
	**VIN**	0.2 ± 0.1	6.0 ± 1.9	**0.002**	N = 15
	**GEM**	1.0 ± 0.4	6.8 ± 2.3	0.063	N = 12
**SCC**					
	**CARB**	1.4 ± 0.6	4.7 ± 1.4	0.084	N = 9
	**VIN**	0.8 ± 0.4	4.9 ± 1.7	0.079	N = 7
	**GEM**	1.3 ± 0.7	4.9 ± 1.7	0.063	N = 7
**Caspase-3**					
**AC**			**RPMI**		
	**CARB**	19.0 ± 4.6	11.1 ± 2.5	0.47	N = 19
	**VIN**	22.0 ± 5.5	10.6 ± 2.4	0.163	N = 20
	**GEM**	20.8 ± 6.4	10.1 ± 2.9	0.559	N = 16
**SCC**					
	**CARB**	13.7 ± 3.2	8.3 ± 1.2	0.483	N = 19
	**VIN**	15.1 ± 3.1	7.8 ± 1.1	0.24	N = 18
	**GEM**	17.5 ± 3.1	8.3 ± 1.4	**0.029**	N = 15

AC: Adenocarcinomas; SCC: Squamous cell carcinomas

RPMI: medium control tissues; CARB: carbolatin; VIN: vinorelbine; GEM: gemcitabine

#### Growth fraction

Table 3 gives an overview of the mean range values (percentage of positively stained cells) obtained for all treatment conditions in AC and SCC separately.

Overall, there was a high variability in the Ki-67 response patterns among the individual NSCLC tissue specimens that appears to be unrelated to histological type of tumor and differentiation grade. However, differences in Ki-67 labeling index which were characteristic for the histological type of NSCLC remained evident under chemotherapy: In the presence of the cytotoxic drugs, Ki-67 expression was markedly inhibited with no significant differences among the individual chemotherapeuticals. In AC, Ki-67 expression was considerably reduced in 88% (14/16), 79% (15/19) and 82% (14/17) of all corresponding tumor samples in response to carboplatin, vinorelbine or gemcitabine, respectively. Furthermore, the remaining percentages of tissue samples consisted of those with negligible Ki-67 <1% already in the medium controls. In marked contrast, SCC revealed reduced Ki-67 expression in only 63% (12/19), 65% (13/20) and 59% (10/17), respectively, of all corresponding tumor samples, demonstrating less responsiveness to the same drugs.

The individual responses in the presence of the cytotoxic drugs carboplatin (CARB), vinorelbine (VIN) or gemcitabine (GEM) as compared to the untreated medium control tissues (RPMI) are also displayed for AC (upper panel) and SCC (lower panel) separately [see Additional files 2 and 6].

A direct comparison of Ki-67 expression (left panel) and BrdU staining patterns (right panel) for the respective treatment conditions is also displayed exemplarily for one human SCC sample as detected by IHC [see Additional files 3 and 6].

#### Apoptosis (cleaved caspase-3)

Overall, the tested NSCLC specimens showed tendencies in the expression patterns of cleaved caspase-3 that loosely can be related to the histological type of lung cancer in response to chemotherapeutical treatment conditions, with AC exhibiting higher expression levels when compared to SCC in response to chemotherapeutical treatment conditions (Table 3). Although all cytotoxic drugs induced expression of cleaved caspase-3 to a similar extent, the results for AC were not statistically significant compared to those of the respective medium controls. The same holds true for SCC; increased expression of this key protein was observed upon treatment with all three chemotherapeutical drugs. Moreover, following treatment with gemcitabine, a statistically significant increase in expression of cleaved caspase-3 was observed compared to the respective medium controls.

For means of completeness, the responses of all individual samples in the presence of the cytotoxic drugs carboplatin (CARB), vinorelbine (VIN) or gemcitabine (GEM) as compared to the untreated medium control tissues (RPMI) are also displayed for AC (upper panel) and SCC (lower panel) separately [see Additional files 4 and 6].

Accordingly, transcription of caspase-3 in four different NSCLC samples (SCC n = 3, AC n = 1) did not exhibit a significant upregulation in response to any of the cytotoxic drugs, which is in marked contrast to the highly responsive SCLC (Fig. 4).

DNA fragmentation was exemplarily examined in human NSCLC specimens that were treated with gemcitabine. The increase in apoptotic cells occurred simultaneously to enhanced expression of cleaved caspase-3, supporting the hypothesis that this protein is suitable as a relevant biomarker for apoptosis. The correlation between these two apoptosis-related parameters in an SCC sample is exemplified in Figs. 9A-D [see Additional files 5 and 6].

#### Association between the observed drug-induced effects (proliferation and apoptosis)

Neither in AC (n = 21) nor in SCC (n = 20) was there any correlation between the expression of Ki-67 and caspase-3: In both cases, only 5 specimens revealed simultaneous alterations that were consistent for each cytotoxic drug. An additional 7 NSCLC samples of both histological types exhibited inversely related changes in response to one or two cytotoxic drugs. In 43% (9/21 AC) and 40% (8/20 SCC) of all tested specimens, however, there were no related patterns of the drug-induced effects.

## Discussion

The major focus of this study was to examine the reliability of a novel *ex vivo *tissue model (STST) and to evaluate multiple aspects of human lung tumor behavior in response to conventional chemotherapy. With such an experimental approach, not only the complexity within the tissue is maintained but also the heterogeneity among individual patients can be studied directly, which was clearly demonstrated by this investigation. STST is a short-term model using comparably small tumor specimens (0.5 g) that are kept in culture for 16 h, which enables high throughput application of many stimuli to a certain lung. This cultivation period was determined to be optimal based on extensive testings in human lung tissue specimens regarding morphology, RNA and DNA integrity [20]. STST was already successfully used for the detection of inducible MCP-1 RNA by RT-PCR in cultivated lung cancer specimens [5]. Furthermore, both induction of the CXCR3 chemokine interferon-gamma-inducible protein-10 (IP-10) [4] and expression of MMP-9 in human lung tissue following *ex vivo *infection with *Chlamydia pneumoniae *were previously demonstrated by *in situ *hybridization in human lung specimens [13]. In a recent report, STST was utilized to elucidate the role of infection with *Chlamydia pneumoniae *within the course of COPD [14].

In a recent publication, an in first instance similar looking approach was undertaken, which was based on formalin-fixed, paraffin-embedded specimens of NSCLC cultivated for 120 h on gelfoam carriers [15]. All molecular read out parameters chosen for apoptosis in this work (caspase-3, TA p73) were described for mitomycin C-treatment; proliferation was measured in untreated specimens but not in chemotherapy-treated tissues. Finally there was no discrimination between adenocarcinomas and squamous cell carcinomas.

The maintenance of the vitality of these tissue samples is crucial for *ex vivo *cultivation. As a consequence of extensive testing the culture period did not exceed 16 h, limiting also the chemotherapeutical treatment to this short-term period that also contributed to the observed moderate effects on programmed cell death. For example, in NCI-H460 NSCLC cells an increase of apoptotic cell death reached approximately 40% at 24 h and 20% at 72 h post-treatment induced by vinorelbine or gemcitabine, respectively [21].

The viability of the human NSCLC tissue samples was only moderately reduced under the chosen culture conditions and did not affect the proliferative capacity of the cells within the tissues as characterized by *de novo *DNA synthesis, when compared to the respective native tumor specimens. Thus, the tumor tissue could appropriately respond to the chemo-therapeutical treatment.

Furthermore, by including samples of chemo-sensitive breast cancer and SCLC, there was a possibility to prove the potential of the chemotherapeutic drugs to induce reduction of proliferation and induction of apoptosis within the chosen conditions. To further validate the STST model, induction of caspase-3 in both chemo-resistant (A 549) and chemo-sensitive cell lines (CPC-N) was compared and revealed the expected difference in sensitivity towards chemotherapy [22]. Proliferation was hardly affected in A549 cells, which could be explained by the resistance of this particular cell line towards chemotherapeutic action. Using other types of cancer cell lines (kidney and cervix), differences in the susceptibility towards cytotoxic drugs were evident [23].

As a first experimental approach to establish STST,*ex vivo *treatment with individual drugs was chosen so that the observed alterations could be unambiguously correlated to the action of a particular drug and give insight into the respective direct drug-tissue interactions. Since the chemotherapeutics were acting towards the same tissue and under identical conditions, direct comparisons of the efficiency of the individual drug could be performed. In NSCLC, the observed drug-induced effects upon a number of multiple biomarkers including Ki-67, BrdU uptake to measure ongoing proliferative activity as well as of cleaved caspase-3 as key protein of the terminal phase in the apoptotic pathway are in good agreement with existing data from both experimental [16,24] and clinical studies [25,26]. However, the consistent differences in the responsiveness of the human NSCLC samples in STST that were closely related to the histological type of tissue have not yet been reported in detail. The demonstrated low correlation between the observed simultaneous drug-induced effects of both cell growth and apoptosis in the individual tissues indicate that the underlying mechanisms are not necessarily linked to each other.

Despite the marked heterogeneity in the responsiveness of the different NSCLC tissue specimens, we not only demonstrated that all three anticancer agents were effective in significantly abrogating proliferation as compared to the untreated tissue cultures but also that gemcitabine was most potent among these conventional chemotherapeutics. Although the alterations relating to apoptosis were more subtle than those for proliferation in IHC, further analyses at the mRNA level and detection of DNA fragmentation as an established parameter for apoptotic cells showed that apoptosis was indeed induced by all tested chemotherapeutical agents to a slightly varying degree.

The chemo-sensitivity of SCLC and breast cancer that was consistently evident following short-term cultivation was in marked contrast to the effects seen in the more chemo-resistant NSCLC specimens, thus revealing the close correlation of our experimental data to the situation *in vivo*. This experimental approach should also be applicable for other types of cancer and it should also be possible to elucidate molecular mechanisms within tumor-free tissues, e.g. the events taking place upon corticosteroid treatment within the human lung.

Based on our results, we conclude that STST is suitable as an *ex vivo *model to study drug-induced effects in lung cancer to provide a base for new strategies of individual and more efficient anticancer treatment for patients with NSCLC.

The complete analysis of the part infiltrating cells might play within the tumor scenario, which would have exceed the extent of this study, is a necessary theme of further investigation. Appropriate studies are underway including the combined chemotherapeutical treatment of the above mentioned conventional drugs as well as selected targeted therapies facing the Epidermal Growth Factor Receptor.

## Competing interests

The author(s) declare that they have no competing interests.

## Authors' contributions

DSL carried out the study, the evaluation and the writing of this manuscript.

DD was involved in the cell culture experiments, flow cytometry and MTT testing.

HS was involved in preparation of the tissues for cultivation and histomorphological evaluation.

DB was responsible for the surgical part of the investigation.

CM and ARR carried out the LIFE/DEAD-assay and 2-photon microscopy.

PZ was involved in the design of the study, evaluation of the results and covered the clinical part.

EV was involved in the design of the study and histomorphological evaluation.

TG conceived the study and was involved in the evaluation of the data as well as the writing of the manuscript.

All authors read and approved the final manuscript.

## Supplementary Material

Additional File 1 Fig. 5 A-B. Immunohistochemical detection of activated caspase-3 in an exemplary human breast cancer tissue sample in the absence (**A**) or presence of gemcitabine (**B**) (all 400×). Click here for file

Additional File 6 Legends. A list of the legends for the additional figures 5-9 is provided Click here for file

Additional File 2 Fig. 6 A-F. Effects of the chemotherapeutic agents carboplatin (CARB), vinorelbine (VIN) or gemcitabine (GEM) on the individual expression of Ki-67 in NSCLC tissues of both adenocarcinoma (upper panel **A-C**) and of squamous cell carcinoma type (lower panel **D-F**) *ex vivo*. The lung tumor specimens were cultivated in medium alone or in the presence of cytotoxic drugs. Alterations of the individual expression patterns of Ki-67 are shown separately in the presence of carboplatin (**A;D**), vinorelbine (**B;E**) and gemcitabine (**C;F**) as compared to the respective untreated medium controls. Click here for file

Additional File 3 Fig. 7 A-H. Comparison of Ki-67 expression (left panel) and BrdU uptake (right panel) determined by IHC in a squamous cell carcinoma in response to the cytotoxic drugs carboplatin (**C;D**), vinorelbine (**E;F**) and gemcitabine (**G;H**). (**A) **(Ki-67) and (**B) **(BrdU) are the respective untreated control tissue samples (all 400×). Click here for file

Additional File 4 Fig.8 A-F. Individual distribution patterns of activated caspase-3 protein in human NSCLC specimens of both adenocarcinoma type (upper panel **A-C**) and of squamous cell carcinoma type (lower panel **D-F) **in the absence (RPMI) or presence of 3 different cytotoxic drugs following 16 h culture period. The results are displayed in accordance to Fig. 5. Click here for file

Additional File 5 Fig.9 A-D. Direct comparison between DNA fragmentation (left panel, A and B) and the expression of activated caspase-3 (right panel, C and D) in apoptotic cells in one exemplary human NSCLC tissue specimen of squamous cell type following gemcitabine. (A) (IHC) and (B) (TUNEL) represent the respective untreated medium control tissues (all 400×). Click here for file
