# Supplementary figures and images for: A novel human ex vivo model for the analysis of molecular events during lung cancer chemotherapy
Source: Respir Res. 2007 Jun 14;8(1):43. doi: 10.1186/1465-9921-8-43 (PMC1913052; doi:10.1186/1465-9921-8-43)

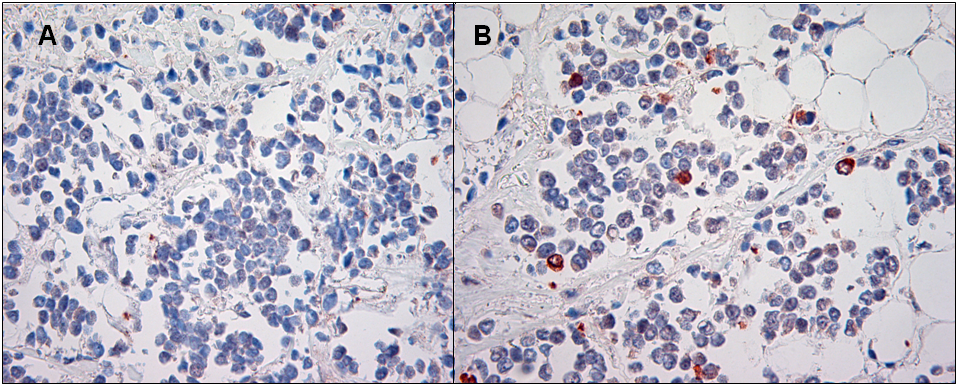

Supplement: Additional File 1 — Fig. 5 A-B. Immunohistochemical detection of activated caspase-3 in an exemplary human breast cancer tissue sample in the absence (A) or presence of gemcitabine (B) (all 400×). [file 1465-9921-8-43-S1.tiff]

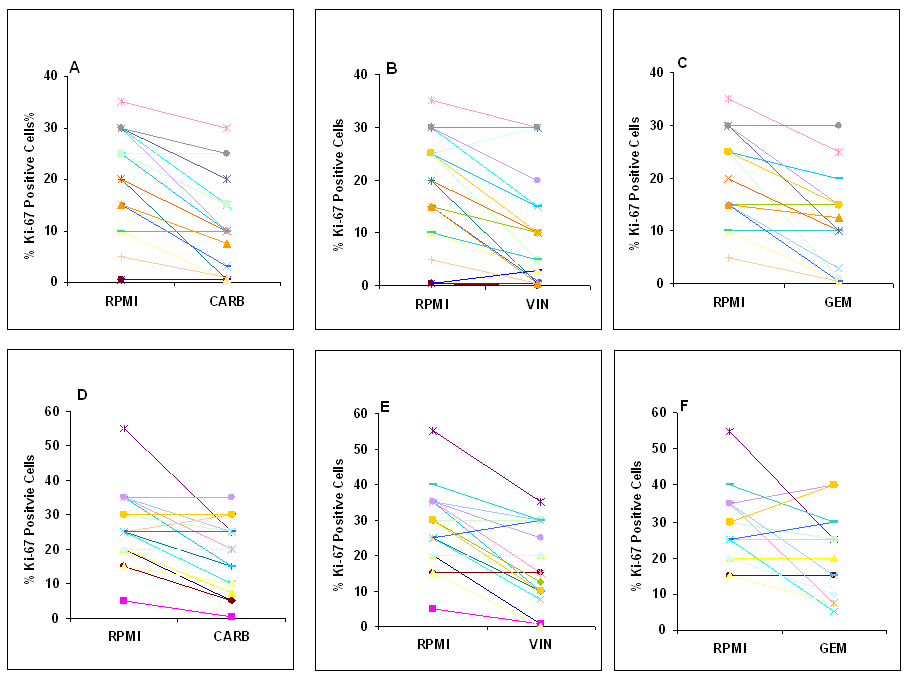

Supplement: Additional File 2 — Fig. 6 A-F. Effects of the chemotherapeutic agents carboplatin (CARB), vinorelbine (VIN) or gemcitabine (GEM) on the individual expression of Ki-67 in NSCLC tissues of both adenocarcinoma (upper panel A-C) and of squamous cell carcinoma type (lower panel D-F) ex vivo. The lung tumor specimens were cultivated in medium alone or in the presence of cytotoxic drugs. Alterations of the individual expression patterns of Ki-67 are shown separately in the presence of carboplatin (A;D), vinorelbine (B;E) and gemcitabine (C;F) as compared to the respective untreated medium controls. [file 1465-9921-8-43-S2.tiff]

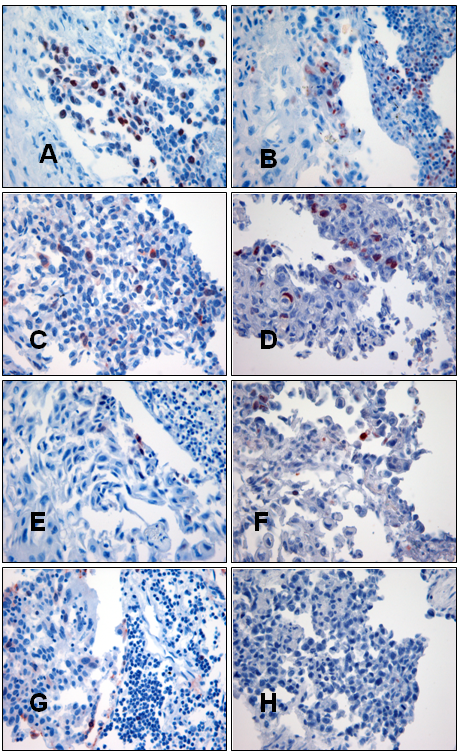

Supplement: Additional File 3 — Fig. 7 A-H. Comparison of Ki-67 expression (left panel) and BrdU uptake (right panel) determined by IHC in a squamous cell carcinoma in response to the cytotoxic drugs carboplatin (C;D), vinorelbine (E;F) and gemcitabine (G;H). (A) (Ki-67) and (B) (BrdU) are the respective untreated control tissue samples (all 400×). [file 1465-9921-8-43-S3.tiff]

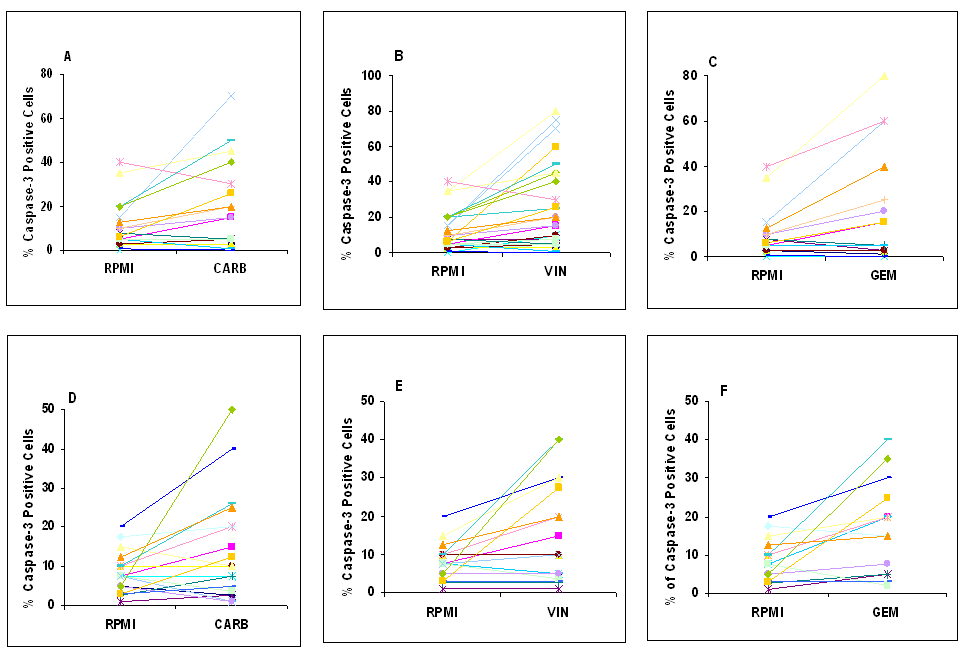

Supplement: Additional File 4 — Fig.8 A-F. Individual distribution patterns of activated caspase-3 protein in human NSCLC specimens of both adenocarcinoma type (upper panel A-C) and of squamous cell carcinoma type (lower panel D-F) in the absence (RPMI) or presence of 3 different cytotoxic drugs following 16 h culture period. The results are displayed in accordance to Fig. 5. [file 1465-9921-8-43-S4.tiff]

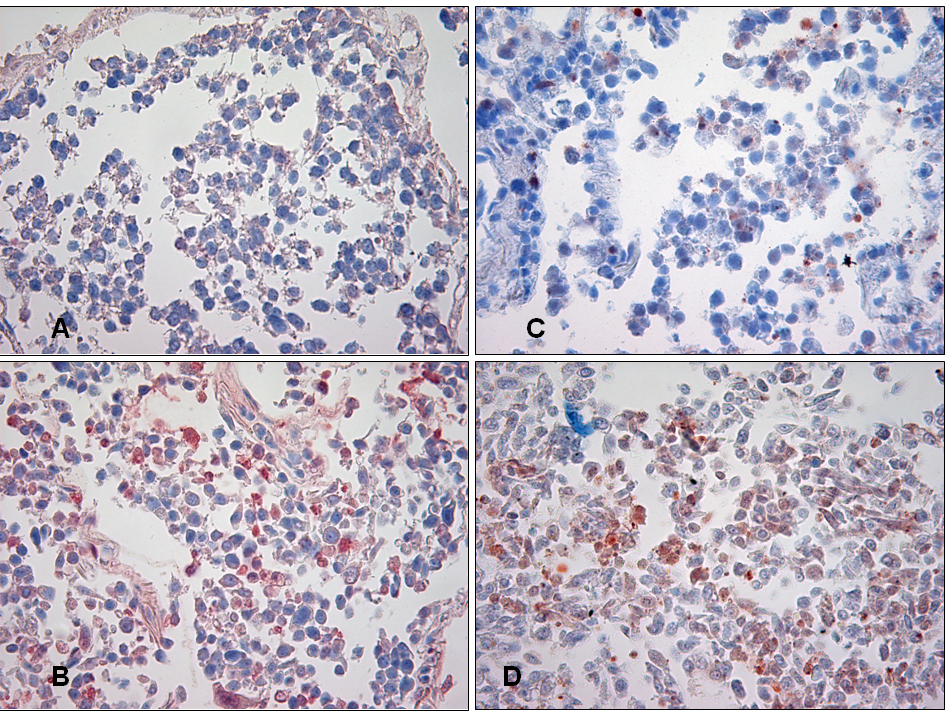

Supplement: Additional File 5 — Fig.9 A-D. Direct comparison between DNA fragmentation (left panel, A and B) and the expression of activated caspase-3 (right panel, C and D) in apoptotic cells in one exemplary human NSCLC tissue specimen of squamous cell type following gemcitabine. (A) (IHC) and (B) (TUNEL) represent the respective untreated medium control tissues (all 400×). [file 1465-9921-8-43-S5.tiff]
