# Supplementary material for: A novel human ex vivo model for the analysis of molecular events during lung cancer chemotherapy
Source: Respir Res. 2007 Jun 14;8(1):43. doi: 10.1186/1465-9921-8-43 (PMC1913052; doi:10.1186/1465-9921-8-43)
Supplement: Additional File 6 — Legends. A list of the legends for the additional figures 5-9 is provided [file 1465-9921-8-43-S6.doc]

## Legends

**Fig. 5 A-B:** Immunohistochemical detection of activated caspase-3 in an exemplary human breast cancer tissue sample in the absence (**A**) or presence of gemcitabine (**B**) (all 400x).

**Fig. 6 A-F**: Effects of the chemotherapeutic agents carboplatin (CARB), vinorelbine (VIN) or gemcitabine (GEM) on the individual expression of Ki-67 in NSCLC tissues of both adenocarcinoma (upper panel **A-C**) and of squamous cell carcinoma type (lower panel **D-F**) *ex vivo.* The lung tumor specimens were cultivated in medium alone or in the presence of cytotoxic drugs. Alterations of the individual expression patterns of Ki-67 are shown separately in the presence of carboplatin (**A;D**), vinorelbine (**B;E**) and gemcitabine (**C;F**) as compared to the respective untreated medium controls.

**Fig. 7 A-H:** Comparison of Ki-67 expression (left panel) and BrdU uptake (right panel) determined by IHC in a squamous cell carcinoma in response to the cytotoxic drugs carboplatin (**C;D**), vinorelbine (**E;F**) and gemcitabine (**G;H**). (**A)** (Ki-67) and (**B)** (BrdU) are the respective untreated control tissue samples (all 400x).

**Fig. 8 A-F**: Individual distribution patterns of activated caspase-3 protein in human NSCLC specimens of both adenocarcinoma type (upper panel **A-C**) and of squamous cell carcinoma type (lower panel **D-F)** in the absence (RPMI) or presence of 3 different cytotoxic drugs following 16h culture period. The results are displayed in accordance to Fig. E1.

**Fig. 9 A-D**: Direct comparison between DNA fragmentation (left panel, A and B) and the expression of activated caspase-3 (right panel, C and D) in apoptotic cells in one exemplary human NSCLC tissue specimen of squamous cell type following gemcitabine. (A) (IHC) and (B) (TUNEL) represent the respective untreated medium control tissues (all 400x).
